# The importance of first impression judgements in interspecies interactions

**DOI:** 10.1038/s41598-020-58867-x

**Published:** 2020-02-10

**Authors:** Laura Clark, Kevin Butler, Kay L. Ritchie, Laëtitia Maréchal

**Affiliations:** School of Psychology, University of Lincoln, Lincoln, UK

**Keywords:** Evolution, Psychology, Animal behaviour

## Abstract

Close human-wildlife interactions are rapidly growing, particularly due to wildlife tourism popularity. Using both laboratory and ecological observation studies we explored potential interspecies communication signalling mechanisms underpinning human-animal approach behaviour, which to date have been unclear. First impression ratings (n = 227) of Barbary macaques’ social and health traits were related to the macaques’ facial morphology and their observed behaviour supporting a shared facial signalling system in primates. These ratings significantly predicted intended approach to the macaques during hypothetical interactions. Finally, real-world interspecies proximity was observed and found to be best predicted by the interaction between human first impression perception and animal behaviour. Specifically, perceived macaque health in interaction with actual macaque dominance drives close interactions despite human proclivity to avoid dominant animals, raising safety concerns in interspecies interactions.

## Introduction

A growing body of evidence suggests that we automatically form first impressions of people from their faces. These first impressions are made quickly^1^, non-consciously^2^, and incidentally without instruction^3^. These first impression judgements have been shown to influence people’s behaviour toward an individual, determining the initiation of positive or negative interactions^4^. For instance, dominance is considered to be a cue of a person’s intentions, and a dominant person could be considered as a potential threat^5^. Although the accuracy of first impression judgements has been largely debated^5–7^, previous studies have shown that first impressions of survival-related traits, e.g. dominance, were more accurate and consistent than more subtle characteristics such as intelligence, suggesting a potential evolutionary advantage of accurate first impression judgements. First impressions are important in interactions as they allow for the prediction of future behaviour^8^, which, if these judgements are inaccurate, could lead humans to put themselves at risk. However, to date, little is known about how humans make judgements of whether and how to interact with individual non-human animals (hereafter animals), and whether these judgements are accurate.

It has been suggested that humans and non-human primates (hereafter primates) have a shared personality facial signalling system, and that both species could use first impression judgements to accurately assess other primate species’ social and health traits^9^. For example, naïve participants have been shown to accurately rate extraversion social traits such as dominance in chimpanzees (*Pan troglodytes*) from static, neutral faces when compared to personality ratings from the chimpanzees’ keepers^9,10^. Japanese and rhesus macaques (*Macaca fuscata* & *Macaca mulatta*) have also been shown to have a visual attentional preference for ‘trustworthy’ human faces^11^. Indeed, morphometric measures of facial structure such as the facial width to height ratio (fWHR) has been established as a reliable cue of dominance and social rank in humans^12^, and primates^13–15^. fWHR has also been shown to be associated with trustworthiness in humans^16^, sex in primates^14^ and aggression in human and other primate species^17,18^. Therefore, if humans can perceive extraversion traits such as dominance, trustworthiness and socialness in primates based on cues of facial structure, it may provide them with insight into the primate’s intentions and potentially significantly impact human willingness to interact with them.

In addition to extraversion traits, humans also make judgements of attractiveness and healthiness based on facial appearance, and these features influence people’s intentions to interact with others^19,20^. For instance, it has been suggested that higher perceived attractiveness is related to higher willingness to approach and positively interact with a person, but also attractiveness is linked to perceived health^19,21^. In interspecies first impression judgements, previous research has shown that humans are able to accurately rate agreeableness/sympathetic traits, and health from neutral chimpanzee faces^10^. In addition, humans have an innate preference for ‘cute’ animals and will prefer to look at and approach ‘cute’ animals whilst avoiding ‘ugly’ animals, possibly due to a perceived risk of contamination^22^. Therefore, perceived attractiveness, cuteness and healthiness judgments might be key to human’s willingness to interact with animals.

Baby schema are cues of ‘cuteness’^22^, defined as a set of infantile features consisting of a round face, large head, big eyes, high forehead, chubby cheeks, small nose and small mouth^23^. The features of a round face and large eyes have also been found to be more typical of a female face^24^. So, a ‘cuter’ face with a high baby schema should be typical of females and this is supported by the established ‘cute is female’ stereotype^25,26^. It has been found that humans are not only sensitive to human infant baby schema but also to baby schema as a rating of cuteness in adult faces^27^. In addition, humans are sensitive to animal baby schema^28,29^ and have an instinctive ability to recognise cuteness in the form of baby schema in animals^30,31^. Therefore, baby schema has the potential to be a valid predictor of cuteness and sex in animals, and might influence human intention to approach/interact with animals.

The rapid expansion of human populations and wildlife tourism popularity have brought humans and wild animals into frequent proximity, which often results in close interspecies interactions. Such close interactions have been shown to increase potential risks to both human and animal welfare^32–34^. For example, primate bites are the second most common animal bite risk to travellers after dog bites, and account for up to 21% of all animal bites worldwide^35^. Despite the potential risks, humans often participate in interactions by either approaching and observing, feeding or taking a photograph with animals^36^. These behaviours are particularly common with primates, such as the Barbary macaque^34,37,38^, yet little is known about the interspecies communication mechanisms underlying human-animal interactions.

This research aims to test for the first time different aspects of first impression formation between species by exploring (1) whether human social trait judgements of Barbary macaques influence intended proximity for approaching, feeding or taking a photograph with the macaques; (2) whether these judgements are based on macaque facial morphological features; (3) if these judgements accurately reflect the real behaviour of the macaques. Finally, this research will explore (4) whether human first impression judgements and/or macaque behaviour best predict human-macaque proximity during real interactions. We hypothesise that human-made social, demographic and health trait judgements will be related to macaque facial morphology and will predict intended proximity and observed macaque behaviour. More specifically, human made dominance judgments will positively relate to fWHR and a high fWHR will negatively predict intended approach. Whilst cuteness, attractiveness and healthiness judgements will positively relate to baby schema with high baby schema positively predicting intended approach. In addition, we hypothesise that both human social trait judgements and observed animal behaviour will be important determinants of proximity in real-life interactions. We conducted an online questionnaire asking 227 participants to rate 17 neutral macaque faces on nine individual characteristics (dominance, trustworthiness, attractiveness, cuteness, healthiness, socialiness, activity, age and sex). We then asked how close participants would get to simply approach, feed and take a selfie with the macaques. In addition to the questionnaire, we also measured each macaque’s fWHR and baby schema based on the pictures. Finally, we used behavioural and human-macaque proximity data previously collected for the 17 macaques shown in the questionnaire.

## Results

**Figure 1:**
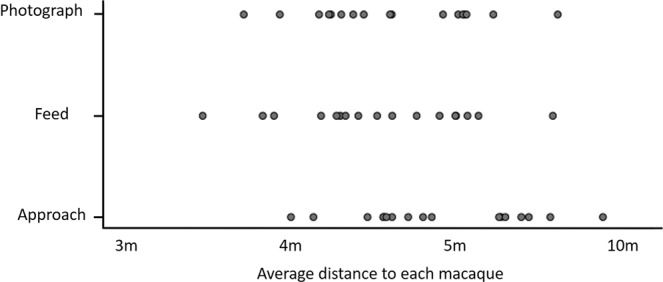
Distribution of the average distance in meters participants reported being willing to approach each macaque for the three conditions: approach, feeding or taking a photograph.

Participants were more likely to report that they would choose to come in to greater proximity to feed the macaques than to take photographs with the macaques (N = 17, t = −2.871, P = 0.011) or simply approach them (N = 17, t = −9.904, P < 0.001, Fig. 1). In addition, participants reported they would be willing to get closer to take a photograph with the macaques than they would to simply approach them (N = 17, t = −11.293, P < 0.001).

*Question 1: Do human social, demographic and health trait judgements of Barbary macaque faces predict intended human-macaque proximity?*

From the online questionnaire, perceived social, demographic and health traits predicted how closely people would approach the macaques to interact with them (Table 1). People were more likely to approach in general, to feed or take a photograph with the macaques when they were perceived as trustworthy, subordinate, cute, social, young and female. In addition, people tended to be more likely to approach the macaques to feed them when they were perceived as healthy, but this did not reach significance.

**Table 1:** Perceived primate social features predicting the type of human-macaque interactions.

Predictor		Approach		Feed		Photograph	
			*P* value		*P* value		*P* value
Full vs. Null model	N	3859	**<0.001**	3859	**<0.001**	3859	**<0.001**
	df	7		7		7	
	L ratio	1515.523		1374.314		1079.064	
(Intercept)	Estimate	0.000	1.000	0.000	1.000	0.000	1.000
	std Error	0.050		0.052		0.055	
	t-value	0.000		0.000		0.000	
Trustworthiness	Estimate	−0.154	**<0.001**	−0.159	**<0.001**	−0.109	**<0.001**
	std Error	0.011		0.011		0.011	
	t-value	−13.783		−14.372		−10.379	
Dominance	Estimate	0.108	**<0.001**	0.107	**<0.001**	0.078	**<0.001**
	std Error	0.011		0.011		0.010	
	t-value	10.074		10.037		7.682	
Cuteness	Estimate	−0.186	**<0.001**	−0.170	**<0.001**	−0.154	**<0.001**
	std Error	0.015		0.014		0.014	
	t-value	−12.733		−11.729		−11.171	
Attractiveness	Estimate	0.010	0.496	0.024	0.097	0.008	0.589
	std Error	0.015		0.015		0.014	
	t-value	0.681		1.660		0.541	
Healthiness	Estimate	−0.005	0.676	−0.023	0.054	−0.015	0.196
	std Error	0.012		0.012		0.011	
	t-value	−0.418		−1.931		−1.294	
Socialness	Estimate	−0.090	**<0.001**	−0.077	**<0.001**	−0.064	**<0.001**
	std Error	0.012		0.012		0.011	
	t-value	−7.585		−6.547		−5.707	
Active	Estimate	0.015	0.196	0.009	0.420	0.005	0.676
	std Error	0.012		0.012		0.011	
	t-value	1.293		0.807		0.417	
Age	Estimate	0.023	**0.019**	0.022	**0.024**	0.026	**0.005**
	std Error	0.010		0.010		0.009	
	t-value	2.341		2.261		2.783	
Sex	Estimate	0.027	**0.004**	0.022	**0.019**	0.022	**0.013**
	std Error	0.009		0.009		0.009	
	t-value	2.856		2.346		2.491	

*Question 2: Are social, demographic and health judgements based on macaque facial morphology?*

Participants seemed to base their social and health judgements on cues found in macaque facial morphology. fWHR was positively correlated with perceived dominance, and negatively correlated with perceived trustworthiness, cuteness and socialness (Table 2). Baby schema was only positively correlated with perceived healthiness. Baby schema was high in perceived females (N = 17, z = −3.621, P < 0.001), but there was no difference in fWHR between sexes (N = 17, z = −0.686, P = 0.492). Perceived age was not correlated to any morphological measures (Baby schema: r_s_ = −0.289, CI 95% = −0.750, 0.228, P = 0.260, fWHR: r_s_ = 0.284, CI 95% = −0.280, 0.697, P = 0.269).

**Table 2:** Relationships between perceived macaque social traits and facial morphological measures.

Perceived social traits		Trustworthiness	Dominance	Cuteness	Healthiness	Socialness
fHWR (N = 17)	r_s_	−0.645	0.510	−0.646	0.208	−0.632
	CI 95%	−0.862, −0.240	0.016, 0.837	−0.873, −0.200	−0.370, 0.744	−0.848, −0.233
	P value	**0.006**	**0.038**	**0.005**	0.421	**0.007**
Baby schema (N = 17)	r_s_	−0.395	−0.022	−0.028	0.510	0.326
	CI 95%	−0.731, 0.089	−0.586, 0.475	−0.552, 0.596	−0.036, 0.876	−0.242, 0.753
	P value	0.118	0.936	0.914	**0.038**	0.201

*Question 3: Do social, demographic and health judgements accurately reflect the real behaviour of the macaques?*

Perceived trustworthiness, cuteness and socialness were negatively correlated with observed aggression rates, while perceived dominance was positively correlated with observed aggression rates (Table 3). Perceived healthiness was negatively correlated to self-scratching rates (r_s_ = −0.708, CI 95% = −0.871, −0.333, P = 0.002). Perceived sex was not significantly related to macaque sex (N = 17, z = −1.667, p = 0.096).

**Table 3:** Relationships between perceived macaque social traits and observed macaque behaviour.

Perceived social traits (First impressions)		Observed macaque behaviour	
		Dominance (N = 17)	Aggression rates (N = 17)
Trustworthiness	r_s_	−0.100	−0.745
	CI 95%	−0.661, 0.532	−0.905, −0.427
	P value	0.701	**0.001**
Dominance	r_s_	−0.284	0.547
	CI 95%	−0.755, 0.342	0.103, 0.764
	P value	0.268	**0.025**
Cuteness	r_s_	0.189	−0.623
	CI 95%	−0.421, 0.746	−0.788, −0.311
	P value	0.468	**0.008**
Socialness	r_s_	0.254	−0.673
	CI 95%	−0.321, 0.717	−0.861, −0.376
	P value	0.326	**0.003**

*Question 4: Which human and/or macaque factors best predict observed human-macaque proximity?*

**Figure 2:**
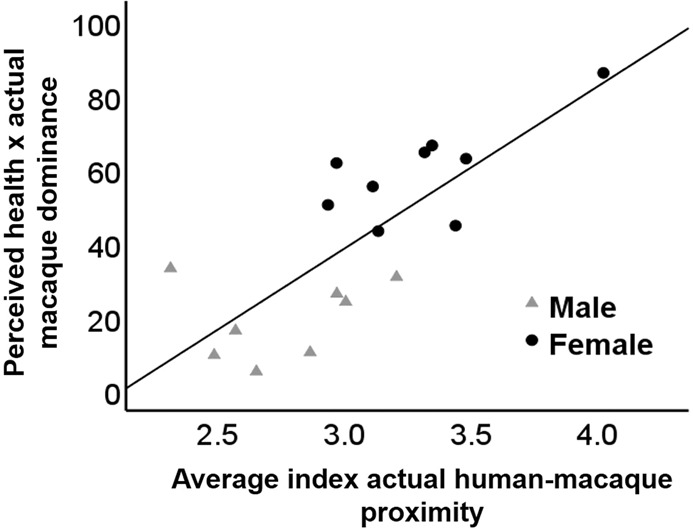
Relationship between the interaction of perceived health and actual macaque dominance and observed human-macaque proximity by macaque sex (male and female).

There was no significant correlation between intended approach behaviour rated by our participants and observed human-macaque proximity (N = 17, r_s_ = −0.240, CI 95% = −0.750, 0.329, P = 0.352). Therefore, human intended proximity with macaques does not singly determine the distance between humans and macaques when interacting. However, macaque dominance predicted the distance between humans and macaques (R^2^ = 0.506, df = (2, 16) = 15.335, *P* = 0.001). Dominant macaques were more likely to be in greater proximity to humans than subordinates (β = 0.711, t = 3.916, P = 0.001). Aggression and self-scratching rates were not predictor of human-macaque proximity during real interactions (aggression: R^2^ = 0.008, df = (2, 16) = 0.116, *P* = 0.738; self-scratching: R^2^ = 0.220, df = (2, 16) = 0.168, *P* = 0.058). Moreover, the interactions between perceived health and observed macaque dominance (β = 0.776, t = 4.761, P < 0.001; Fig. 2) best predicted the observed distance between humans and macaques (R^2^ = 0.602, df = (2, 16) = 22.669, *P* < 0.001).

## Discussion

Close human-wildlife interactions increase risks to both human and animal welfare. Therefore, being able to make an accurate judgement of survival-related social, health and demographic characteristics of wild animals would lead to an effective interspecies communication, which could reduce the risks for both species. Overall our findings support our hypothesis that human first impression judgements related to intended approach to primates. Our results indicate that people are more likely to intend to approach, approach to feed or approach to take a photograph with a macaque when the macaque was perceived as trustworthy, subordinate, cute, social, young and female. Facial morphology measures were highly correlated to social characteristics and sex, which suggest that humans may use these morphological clues to determine these characteristics in macaques. In addition, perceived macaque social characteristics were highly correlated with observed macaque aggression rates, perceived health was negatively correlated with observed macaque self-scratching rates- an indicator of animal welfare. Finally, although people seem to be able to accurately assess survival-related characteristics in macaques based on pictures of their neutral facial expression, and the perceived characteristics predict their intended proximity to each macaque; this is not related to observed human-macaque proximity recorded in the real world. The interactions between perceived macaque health and actual macaque dominance were the best predictors of the observed distance between both species.

Our findings support the hypothesis that humans and other primate species share a social and health facial signalling system^39^, that is used by humans to determine intended approach to macaques. Humans intended to closely interact with macaques that they perceived as trustworthy, subordinate, cute, social, young and female. These perceived social and demographic traits influenced the distance they would be willing to approach each macaque independent of the type of interaction, observation, feeding or taking a photograph. These are the same characteristics preferred for human-human interactions. People are more likely to approach and interact with someone perceived as trustworthy, subordinate, attractive, social, young, and female^26,40–42^. Interactions with a person presenting these characteristics is thought to be preferred because they are ‘safer’ with lower risk of conflict^5^. Humans might also prefer these characteristics to reduce possible health risks when closely interacting with another primate species, or more broadly another animal species.

As previously mentioned, humans have a preference to look at ‘cuter’ animals^22^. For instance, hikers were more likely to stop and approach and take photographs with animals they viewed as ‘cute’^43^. The authors explain that this behaviour was due to the ‘cute response’, which they described as ‘the compulsion to nurture and not hurt the cute entity’. This so-called ‘cute response’ has also been linked to anthropomorphism by transposing human societal values onto animals^44,45^. The anthropomorphic views of wildlife as being ‘cute’ removes the concept of unpredictability in potentially dangerous wild animals^46^, meaning humans will not perceive approaching ‘cuter’ animals as dangerous or risky so they will be more inclined to do so.

Our results indicate that perceived social and health traits were significantly correlated to observed macaque behaviours, and thus suggest that humans can accurately assess primate’s social traits and health status based on their faces. Trustworthiness, cuteness and socialness were negatively correlated to observed rates of macaque aggression, but perceived dominance was positively related to observed rates of macaque aggression. No perceived social, health or demographic traits were related to observed macaque dominance. Perceived health was negatively associated with observed rates of macaque self-scratching, a proxy of animal welfare^33^. However, humans were not able to assess the sex of the macaques. No actual record of macaque age was available to determine the accuracy of perceived age. Previous studies have found that humans are able to accurately assess dominance, trustworthiness, agreeableness (here: cuteness), healthiness, socialness, age but not sex in chimpanzees^9,10^. In addition, human ability to accurately assess these traits in faces in different primate species might explain the high correlation found between trait ratings given by familiar observers and behavioural coding methods (e.g., rhesus macaques^47^; vervet monkeys, *Chlorocebus pygerythrus*^48^; chimpanzees^49,50^).

Our findings also demonstrate that facial morphology in macaques (fWHR and baby schema) can transmit similar social, health and demographic information as do human faces, and that humans can identify and use this information when deciding to approach macaques. Indeed, higher fWHR has been negatively related to trustworthiness and positively associated with dominance in many primate species including humans^4^, capuchins, *Sapajus Cebus sp*^14^, macaques, *Macaca* genus^18^. Recently, Japanese and rhesus macaques have been shown to display preferential attention toward human faces with lower fWHR, suggesting that macaques can form human like first impressions based on facial features^11^. Therefore, it appears that both macaques and humans may share the cognitive mechanisms required for processing social and health traits from facial morphology, supporting the idea of an honest interspecies communication.

Contrary to our predictions baby schema measurements were not correlated with perceived cuteness or age. Perceived cuteness is highly associated with age in animals, with younger individuals perceived as cuter than adults^31,51^. The age variability in our macaques was limited as all macaques were adults, which may explain why the baby schema measurements did not correlate with perceived age nor perceived cuteness. However, baby schema measurements were positively correlated with perceived health and sex. Previous research has shown that baby-faced adult humans were judged as less dominant, more honest, feminine and approachable^24,52,53^. Therefore, such facial morphological clues might be used by humans to assess health and sex in macaques, and such clues influence intended approach proximity towards the macaques. However, perceived sex was not related to sex in macaques, which can be explained by the low sexual dimorphism in Barbary macaque neutral faces.

Although our findings support our hypothesis that human first impression judgements related to intended approach, intended behaviour made by our participants did not reflect observed proximity between humans and macaques observed at the field site over an 11-months period. Actual macaque dominance in interaction with perceived health best predicted the observed distance between humans and macaques (over 60%). Given that our findings suggest that humans intend to approach subordinate animals more closely, this suggests that macaques play an active role in human-macaque interactions, with dominant individual macaques approaching more closely than humans intend or perceive to be safe. Previous studies have found that dominant primates have precedence over clumped food resources, and conflicts often occurs between macaques when food distribution is spatially limited^54,55^. As most human-macaque interactions at the field site were driven by food^38^, the greater proximity of dominant macaques compared to subordinate individuals is therefore expected. In addition, perceived health seemed to influence the proximity to which the humans interact with individual macaques. Although perceived health appears to accurately reflect overall health status of macaques, it appears that human-macaque interaction rates do not significantly change when macaques are ill as reported in^56^ using the same macaque group as in this study. This highlights serious concerns for potential pathogen transmission between both species.

Although intended approach to each macaque was not related to observed human-macaque proximity recorded in the real-world it is important to consider some further points in relation to this finding. Firstly, in the real-world additional cues such as different and changing macaque facial expressions, body gesture and vocalisation, not only neutral faces can be used to guide macaque approachability. In addition, observed human approach attempt could have been more representative of the intended approach measured in the questionnaire than observed proximity, which is co-dependent of both human and animal behaviour. However, such measure was not available. Therefore, future research should investigate the relationships between human approach attempt and achieved proximity to better dissociate the influence of the human first impression judgements from the macaque behaviour. Also, intended or observed interspecies proximity may be associated with human traits such as risk-taking, sensation seeking and neuroticism. However, to date, none of these factors have been studied in relation to wildlife approachability and further research should be conducted to establish the relative importance of these factors in interspecies interactions. Finally, it should be noted that intended approach in this analysis was the aggregate from 227 individuals and that observed proximity for real-life interactions was determined from a different group of individuals potentially masking a significant correlation between intended and observed proximity.

In conclusion, our results provide evidence of a shared facial signalling system in primates. Such honest facial signalling may be an evolutionary adaptation that confers advantages in intra- and interspecies interactions, e.g. preventing potentially detrimental conflicts or the spread of pathogens. Such information is used by humans to make accurate judgements of social and health traits of macaques, resulting in an adjustment of their intended behaviour towards the macaques. However, an inter-species interaction requires the involvement of at least two parties. Here our findings suggest that feeding wildlife increases the risks to human safety by bringing dominant animals in to greater proximity with humans, who would otherwise be less likely to intend to approach these animals based on their first impression judgement. We suggest that regulating the feeding of wildlife is an urgent requirement to reduce human and animal welfare risks.

## Methods

### Participants

Two hundred and twenty-seven participants took part (37 males, 189 females & 1 non-binary), aged between 18 and 76 years (M = 24.9 years, SD = 13.3 years). All aspects of the data collection and analysis reported were carried out in accordance with the guidelines approved by the University of Lincoln School of Psychology ethics committee (ethics code PSY1718540).

### Procedure

The study was conducted online via Qualtrics software (Qualtrics2018 v. 8). The link to the study was distributed to students at the University of Lincoln in exchange for course credits, and to the general public via social media. Participants then rated faces of 17 Barbary macaques on seven social traits (dominance, trustworthiness, attractiveness, cuteness, healthiness, socialness and activity) on a 7-point Likert scale from extremely low to extremely high. Demographic-like traits of age and sex were rated on a 7-point Likert scale from very young to very old and very feminine to very masculine. Participants saw each monkey face individually, and rated each face on all nine traits before moving on to the next face. Participants were then asked to select how close they would get to approach, approach to feed or approach to take a selfie with each monkey; from 11 options ranging from 0 m to 100 m, including an option to not approach.

### Stimuli

The images used were all faces of different Barbary macaques from the same group, 8 males and 9 females taken by the corresponding author. All images showed Barbary macaques with neutral facial expressions, in a frontal pose, and in high resolution to allow facial morphological measurements.

#### *Measurements*

**Figure 3:**
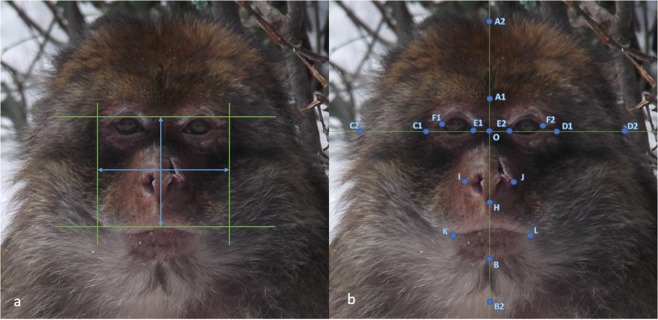
Facial landmarking guides (example: Barbary Macaque Pe1). Image A demonstrates the positioning of bizygomatic width and upper face height, required for measuring fWHR. Image B shows the facial landmarks required for measuring baby schema. The green lines act as guides as it is the length of the blue lines or distance between the blue dots that were measured.

All facial measurements were taken using the image manipulation programme GIMP 2.10.4, including the measure tool and rectangle select tool (Fig. 3). Each macaque image was scaled to a width of 130 mm and measurements were collected in mm and recorded to two decimal places. All measurements were taken by two independent researchers. All measurements were within 10 mm of one another, so all were accepted, and the final measurement used for the following calculations was the average of both measurements.

#### *Measuring the facial width to height ratio (fWHR)*

Facial width to height ratio (fWHR) was calculated from the maximum horizontal distance from the left to right zygion referred to as bizygomatic width and the upper face height, measured as the vertical distance from the upper lip to the highest point of the eyelids^14,18^ (Fig. 1). Using the GIMP 2.10.4 rectangle select tool, a box was drawn out to these specifications and then measured accordingly. The final measurement was calculated by bizygomatic width divided by upper face height.

#### *Measuring baby schema*

Baby schema measurements were adapted from two papers^23,31^. The distance between selected facial landmarks were measured (Fig. 1): Forehead length (AO1), Left eye width (E1F1), Right eye width (E2F2), Average eye width (EF; calculated from E1F1 and E2F2), Face width (CD1), Face length (AB1), Mouth length (KL), Nose length (OH), Nose width (IJ). Using these measurements, five facial parameters were required to calculate baby schema: AO/AB1, EF/CD1, OH/AB1, IJ/CD1, KL/CD1. The results of these calculations were all then scaled appropriately so that AB1 equalled 100 mm, to control for the variance of facial height among each image. The mean and standard deviation for each facial parameter were then calculated. Comparing each scaled measurement to its mean, if AO/AB and EF/CD were greater than the mean it showed high baby schema. If OH/AB, IJ/CD and KL/CD were less than the mean it showed high baby schema. If the facial feature indicated high baby schema it stayed a positive number. If it indicated low baby schema, it was changed to a negative number, for example from 0.5 to −0.5. Final baby schema scores were the average of each facial parameter z scores.

### Observed macaque behavioural and human-macaque interaction data

This work followed the Animal Behaviour Society’s guidelines for the treatment of animals in behavioural research and teaching, adhered to standards as defined by the European Union Council Directive172 86/609/EEC. Research permission was provided by the Haut-Commissariat aux Eaux et Forêts et à la Lutte Contre la Désertification of Morocco (Number 235). Data were collected from February-December 2012 on 17 adult Barbary macaques; all from one group experiencing daily tourist interactions, and located in Ifrane National Park, Morocco (33°25.0N; 005°10.0W). Continuous focal sampling^56^ was used to assess average rates of aggression and self-scratching, and scan sampling was used to measure the distance between humans and macaques such as 0 m, 0–1 m, 1–2 m, 2–5 m, and 5–10 m (for more details^38,57^). The dominance rank of each macaque was calculated using corrected normalized David’ scores based on the outcomes of all visible same-sex dyadic conflicts with no counter-aggression using *ad libitum* sampling^38^.

### Data analysis

Prior to analysis, the perceived trait, age and sex data were ranked from lowest score (1) to highest score (7), and an index of distance was used ranging from touching (0) to would not approach (11). All categorical variables were then z-transformed to improve the interpretability of the variables^58^. Question 1: A series of GLMMs was used to explore whether participants’ willingness to approach, approach to feed the macaques and approach to take a photograph with the macaques (dependent variables) were influenced by macaque characteristics (predictors: dominance, trustworthiness, attractiveness, cuteness, health, sociality, activity, perceived age and sex). All models were fitted using R software (R 3.5.3, R development core team 2018). All ranked variables such as index of distance and perceived traits were z-transformed to improve the interpretability of the variables^58^. The three models had as non-nested random factors the identities of the macaques and the participants. The models used the function lme of the R-package nlme for Gaussian linear mixed-effects models^59^. For each model, the significance of the full model was compared to the corresponding null model, i.e. model with all predictions replaced by ‘1’, using a likelihood ratio test (R function ANOVA). All models were checked to assess whether they violated any assumptions, which none did, including collinearity (VIF function, all VIF results <4, ranging 1.3 to 2.2), outliers (Cook’s distance = 0.007, no outlier found), distribution and homogeneity of the residuals^60^.

Questions 2–3: Spearman’s rho correlations (Spearman’s R, using the cor function in R) were then used to investigate the relationships between trait judgement scores and macaques’ facial morphological measurements. A Wilcoxon test (R function wilcox.test) was used to compare perceived sex ranked to macaques’ facial measurements and Chi square test (R function chisq.test) was used to compare binary perceived sex (Female ≤3.5 average scores, Male >3.5 average score) to actual sex of the macaques. No Bonferroni correction was applied as analyses on each variable were hypothesis driven and tested fewer than ten times, as well as to ensure that the likelihood of the type I error was low^61^. These tests were conducted using R (R 3.5.3, R development core team 2018), significance at α = 0.05, two-tailed. All continuous variables were not normally distributed (Shapiro tests *p* < 0.05) or ranked, so non-parametric tests were used.

Question 4: First a spearman correlation was used to explore the relationships between intended behaviour and observed human-macaque proximity per macaque (N = 17). Then, a series of linear regressions (R function lm) was used to determine which human’s first impression judgements (average scores rated by the 227 participants for each macaque) and/or macaques’ behaviour best predict human-macaque proximity during real interactions. A series of simple linear regression were conducted to assess which macaque behaviours best predicted real human-macaque proximity, i.e. dominance, aggression rates and self-scratching rates. Then, as 50% of the variability in proximity was predicted by macaque actual dominance (see result section), interactions between judgment traits and actual dominance were then explored, i.e. cuteness * dominance, health * dominance, age * dominance. Due to the high collinearity between the predictors, several simple linear regressions were conducted rather than one multiple linear regression. Only interactions that increased human-macaque prediction were accepted (R^2^ > 0.506). All regressions were checked to assess whether they violated any assumptions, including outliers, distribution and homogeneity of the residuals^60^.

## Data Availability

Data are available on the repository of the University of Lincoln (https://eprints.lincoln.ac.uk/).
